# Yes-associated protein regulates podocyte cell cycle re-entry and dedifferentiation in adriamycin-induced nephropathy

**DOI:** 10.1038/s41419-019-2139-3

**Published:** 2019-12-04

**Authors:** Kewei Xie, Chenqi Xu, Minfang Zhang, Minzhou Wang, Lulin Min, Cheng Qian, Qin Wang, Zhaohui Ni, Shan Mou, Huili Dai, Huihua Pang, Leyi Gu

**Affiliations:** Department of Nephrology, Molecular Cell Lab for Kidney Disease, Ren Ji Hospital, School of Medicine, Shanghai Jiao Tong University, Shanghai, 200127 People’s Republic of China

**Keywords:** Cell biology, Molecular biology

## Abstract

Podocytes are terminally differentiated cells with little proliferative capacity. The high expression levels of cell cycle inhibitory proteins, including p21, p27, and p57, play an important role in maintaining the low level of proliferation of mature podocytes. In the present study, we aimed to explore the role of yes-associated protein (YAP) signalling in adriamycin-induced podocyte re-entry into the cell cycle and dedifferentiation. Proliferating cell nuclear antigen (PCNA)-, cyclin-dependent kinase 4 (CDK4)-, and Cyclin D1-positive podocytes were found in mice with adriamycin-induced nephropathy. In vitro, adriamycin administration increased the percentage of cells in S phase and the upregulation of mesenchymal-related marker proteins. CDK4 and cyclin D1 were significantly up-regulated after incubation with adriamycin. Overexpression of YAP in podocytes promoted their entry into the cell cycle; up-regulated cyclin D1, desmin, and snail2 expression and down-regulated Wilms’ tumour 1 (WT1) and nephrin production. Recombinant murine FGF-basic induced podocytes to re-enter the cell cycle, inhibited WT1 and nephrin, and increased desmin and snail2 expression. Pretreating podocytes with verteporfin, an inhibitor of YAP/ TEA domain transcription factor (TEAD), decreased the adriamycin-induced overexpression of cyclin D1 and reduced the ratio of S-phase podocytes. This result was further verified by knocking down *YAP* expression using RNA interference. In conclusion, adriamycin induced podocytes to re-enter the cell cycle via upregulation of CDK4 and cyclin D1 expression, which was at least partly mediated by YAP signalling. Re-entry into the cell cycle induced the over-expression of mesenchymal markers in podocytes.

## Background

Proteinuria is the main clinical manifestation of kidney disease and podocyte injury is the cytological basis for the development of large amounts of proteinuria^1^. Podocytes, also known as glomerular visceral epithelial cells, are terminally differentiated cells with little or no proliferative capacity. They cover the glomerular basement membrane and their adjacent foot processes are staggered, forming a key component of the glomerular filtration membrane barrier^2,3^. The maintenance of podocyte function depends on its highly differentiated state. Dedifferentiation of podocytes, caused by various factors, leads to podocyte damage, which ultimately cause podocyte loss, resulting in proteinuria.

Escape from the cell cycle due to the high expression levels of cell cycle inhibitory proteins, including p21, p27, and p57, is the main reason mature podocytes can maintain low levels of proliferation^4^. Recent studies have shown that, in some glomerular diseases, such as focal and segmental glomerulosclerosis^5^, collapsed glomerular disease^6^, and human immunodeficiency virus (HIV)-related nephropathy^7,8^, injured podocytes re-enter the cell cycle. These podocytes have increased susceptibility to pathogenic compounds and express mesenchymal-associated proteins^9^. Until now, little is known about the specific molecular biological mechanism of podocyte re-entry into the cell cycle and its relationship with podocyte dedifferentiation in pathological states.

Embryonic podocytes are derived from mesenchymal-epithelial trans-differentiation and mature podocytes retain some of the characteristics of mesenchymal cells, while expressing epithelial cell marker proteins^10,11^. Various cell damage factors lead to podocyte dedifferentiation, which is manifested by the loss of marker proteins and increased expression of mesenchymal-associated proteins. Mature podocytes are rich in actin cytoskeleton and it has been reported that podocyte re-entry into the cell cycle may lead to changes in the cytoskeleton, thus causing dedifferentiation and loss of protein filtration barrier function^12^. However, the molecular biological mechanisms involved in this process are currently poorly understood.

The Hippo/YAP pathway exerts its effects mainly by regulating the activity of its downstream effector molecule, YAP. This pathway is a kinase chain consisting of a series of protein kinases and transcription factors^13^. Numerous studies have shown that the Hippo pathway plays an important role in kidney development and the inhibition of podocyte apoptosis^14^. In addition, activation of YAP signalling can also upregulate cell cycle-associated protein expression, thereby promoting cell proliferation^15^. Furthermore, YAP signalling is involved in the regulation of TGF-β1 signalling-mediated apoptosis and epithelial-mesenchymal transition^16^. Therefore, this study focused on the role of YAP signalling in podocyte cell cycle regulation and dedifferentiation.

## Results

### Adriamycin promoted podocyte re-entry into the cell cycle

**Fig. 1:**
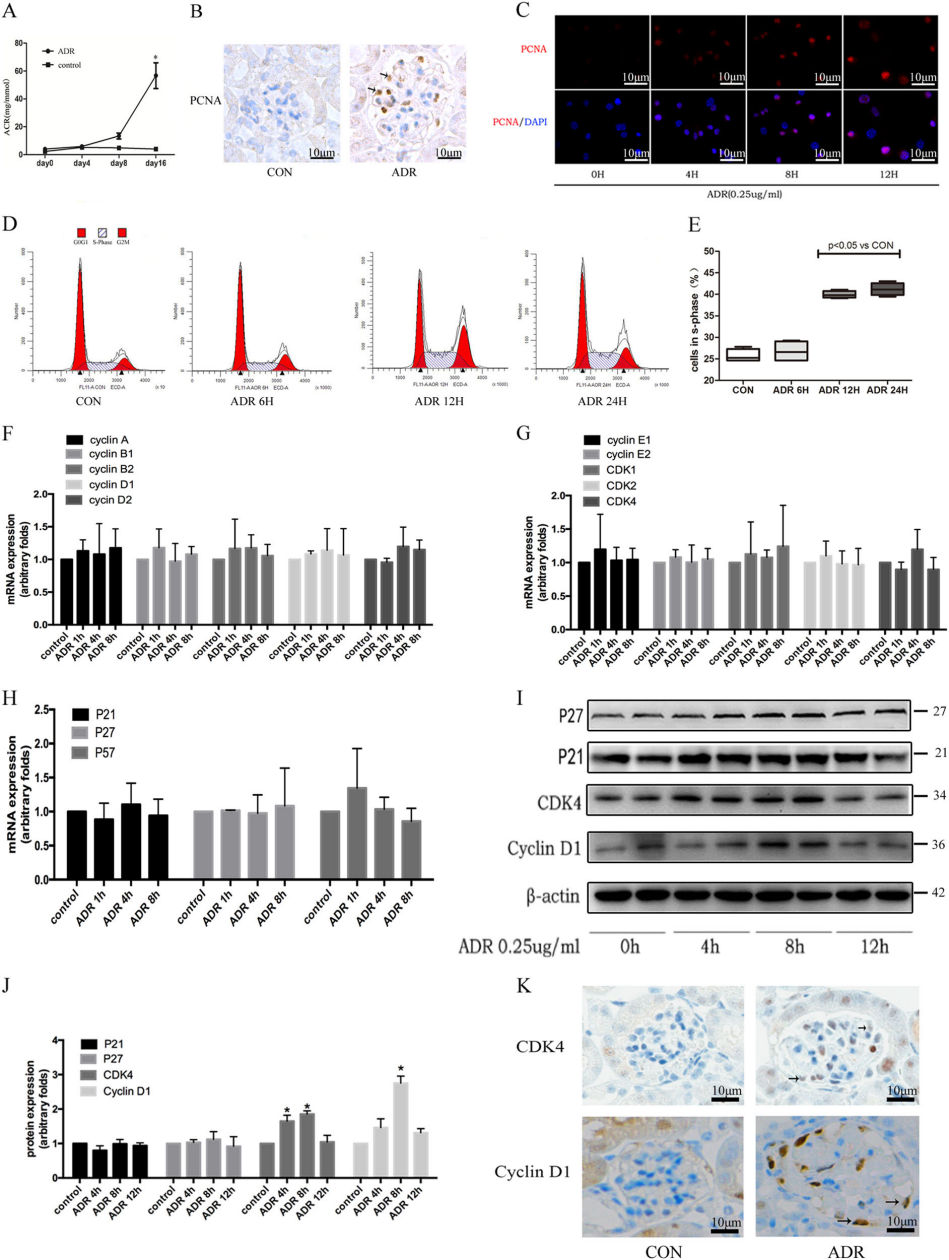
Injured podocytes re-entered the cell cycle. **a** Urinary albumin/creatinine ratio in mice of the control group and adriamycin-treated group; error bars indicate the SD (*n* = 3). **b** Immuno- histochemical staining showed expression of PCNA in the glomerulus; podocytes are indicated by an arrow (magnification, ×200). **c** In vitro experiments showed the expression of PCNA in podocytes after adriamycin (0.25 µg/mL) treatment for the indicated time (magnification, ×200). **d** Schematic diagram of cell cycle detection results; the diagonal strip area represents the proportion of cells in S phase. **e** The proportion of cells in S phase in different groups; data are from at least three replicates. **f**–**h** Real-time PCR detection of cell cycle-related gene expression after adriamycin treatment; data are from at least three replicates. **i** Western blotting analysis of the expression of cell cycle-associated proteins and their inhibitors in podocytes treated with adriamycin (0.25 µg/mL) for the indicated time periods. **j** Quantification of band density relative to the β-actin control (*n* = 3 per group). **k** Immunohistochemical analysis showed that CDK4 and cyclin D1 were expressed in podocytes of adriamycin-treated mice; the arrow indicates the location of the podocyte and the magnification was ×400. *CON* control, *ADR* Adriamycin, *DAPI* 4′,6-diamidino-2-phenylindole. *P < 0.05 vs. control group.

In vivo, we found that the urinary albumin/creatinine ratios of the control group at 0, 4, 8, and 16 days were 2.3 ± 0.18 mg/mmol, 5.2 ± 0.39 mg/mmol, 4.8 ± 0.07 mg/mmol, and 4.05 ± 0.44 mg/mmol, respectively, while the urinary albumin/creatinine ratios in adriamycin-treated mice at 0, 4, 8, and 16 days were 4.3 ± 1.18 mg/mmol, 5.3 ± 1.05 mg/mmol, 13.4 ± 3.6 mg/mmol, and 56.6 ± 16.0 mg/mmol. After 16 days of adriamycin administration, mice developed significant proteinuria. Immunohistochemical staining revealed proliferating cell nuclear antigen (PCNA) expression in some podocytes of adriamycin-treated mice, suggesting that some podocytes had entered S phase (Fig. 1b). In vitro, after adriamycin stimulation of podocytes for 4 h, 8 h, and 12 h, the PCNA expression was significantly increased (Fig. 1c). We used adriamycin treatment to stimulate the differentiation of podocytes, as shown in Fig. 1d, e. Flow cytometric analysis showed that these podocytes re-entered the cell cycle, with 26.52 ± 0.65%. 28.28 ± 0.57%, 39.94 ± 0.84%, and 41.18 ± 1.46% of cells entering S phase after 0, 6, 12, and 24 h of treatment, respectively. Compared with the control group, a significantly greater proportion of cells were in S phase after 12 and 24 h of adriamycin treatment (*P* < 0.05).

### Injured podocytes expressed CDK4 and cyclin D1

We further explored cell cycle-associated regulatory gene expression and protein production. As shown in Fig. 1f–h, the mRNA expression of cyclins; the protein kinases, *CDK 1*/*2*/*4*; and their corresponding inhibitory regulators were not significantly changed, compared with the control group, after 1, 4, or 8 h of adriamycin treatment. At the protein level, the expression of cyclin D1 began to increase after 8 h of adriamycin treatment, reaching 171.99 ± 16.56% of the expression levels in the control group (*P* < 0.05). After adriamycin treatment of podocytes in vitro for 4 and 8 h, CKD4 protein expression was up-regulated by 60.08 ± 19.1% and 92.04 ± 9.28% respectively, compared with the control group. The expression of both proteins returned to baseline levels after 12 h, while the expression of the CDK inhibitor proteins, p21 and p27 showed no significant changes with adriamycin treatment (Fig. 1i, j). Immunohistochemical staining revealed the overexpression of cyclin D1 and CDK4 in podocytes from adriamycin-treated mice (Fig. 1k).

### Cell cycle re-entry sensitised podocytes to dedifferentiation

**Fig. 2:**
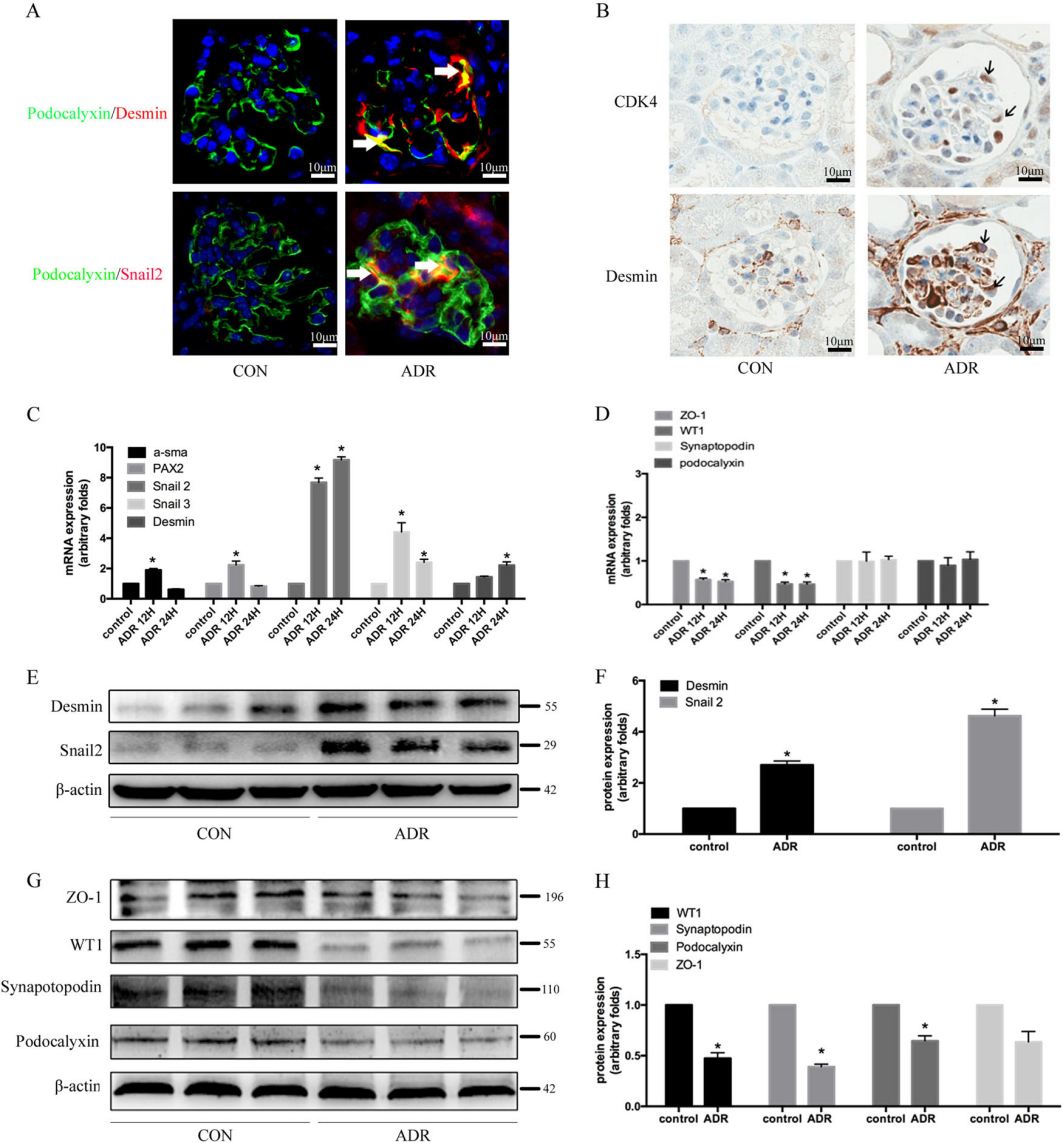
Cell cycle re-entry sensitised podocytes to dedifferentiation. **a** Immunofluorescence staining for the detection of snail2 and desmin expression in mouse kidneys; the arrow indicates co-staining of snail 2/desmin (red) and podocalyxin (green) in podocytes (magnification, ×400). **b** Immunohistochemical staining showed that some CDK4-positive podocytes in adriamycin-treated mice began to express desmin. The arrow indicates podocytes expressing CDK4 and desmin simultaneously (magnification, ×400). **c**, **d** mRNA expression of dedifferentiation-related genes and podocyte markers examined by real-time PCR after adriamycin treatment for the indicated time periods. **e** The effect of adriamycin on the expression of snail2 and desmin was determined by western blotting analyses. **f** Immunoblotting quantification of (E). **g** Western blotting analysis of podocyte marker protein expression after adriamycin (0.25 µg/mL) treatment for the indicated times. **h** Immunoblotting quantification of (G). Error bars are ± SD. (*n* = 3 per group). *P < 0.05 vs. control group.

We further explored the changes in the biological functions of podocytes after re-entering the cell cycle. As shown in Fig. 2a, some podocytes from adriamycin-treated mice simultaneously expressed desmin and snail2. To explore the relationship between cell cycle re-entry and podocyte dedifferentiation, serial immunohistochemical staining sections showed CDK4 and desmin double-positive podocytes, indicating that podocytes entering the cell cycle had begun to dedifferentiate (Fig. 2b). An in vitro study was performed to stimulate differentiated podocytes with adriamycin. As shown in Fig. 2c, d, the expression levels of the dedifferentiation-related genes, *α-sma*, *PAX2*, *snail2*, and *snail3* increased by 95.12 ± 5.08%, 102.31 ± 10.04%, 713.24 ± 10.15%, and 354.68 ± 20.39%, respectively, after 12 h of treatment. The expression of these genes returned to basal levels after 24 h, with the exception of *snail2* and desmin expression, which continued to rise. Expression of the podocyte marker genes, *ZO-1* and *WT1*, decreased at the mRNA level by 45.78 ± 5.12% and 59.13 ± 5.01%, respectively, after adriamycin administration for 12 h and by 49.32 ± 6.08% and 60.12 ± 7.08% respectively, after 24 h compared with control group, while adriamycin had no significant effect on the expression of synaptopodin or podocalyxin. At the protein level, the expression of the podocyte markers, WT1, podocalyxin, and synaptopodin were found to be reduced by 51.08 ± 6.12%, 24.13 ± 6.04%, and 61.19 ± 4.71% compared with the control group after 48 h of adriamycin administration, while the expression of the dedifferentiation-related proteins, desmin and snail2, were increased by 135.82 ± 8.91% and 322.56 ± 12.92%, respectively (Fig. 2e–h).

**Fig. 3:**
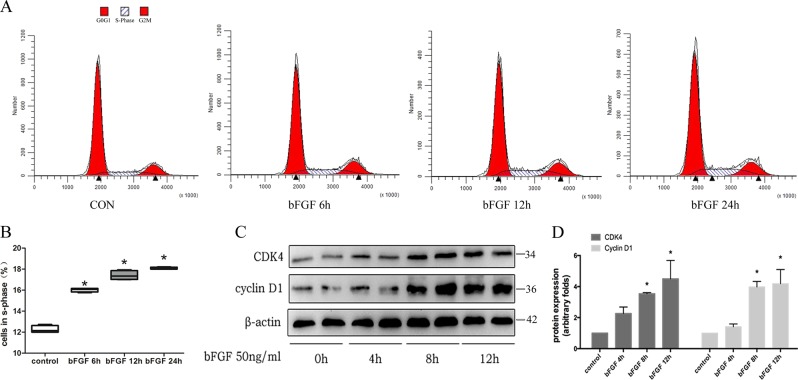
bFGF promoted podocyte re-entry into the cell cycle. **a** Schematic diagram of cell cycle detection results after bFGF treatment for the indicated time periods. **b** The proportion of cells in S phase in different groups; data are from at least three replicates. **c** Western blotting analysis of CDK4 and cyclin D1 protein expression after bFGF (50 ng/mL) treatment for the indicated times. **d** Corresponding graph of (C); each bar represents data obtained from there independent experiments. *P < 0.05 vs. control group.

**Fig. 4:**
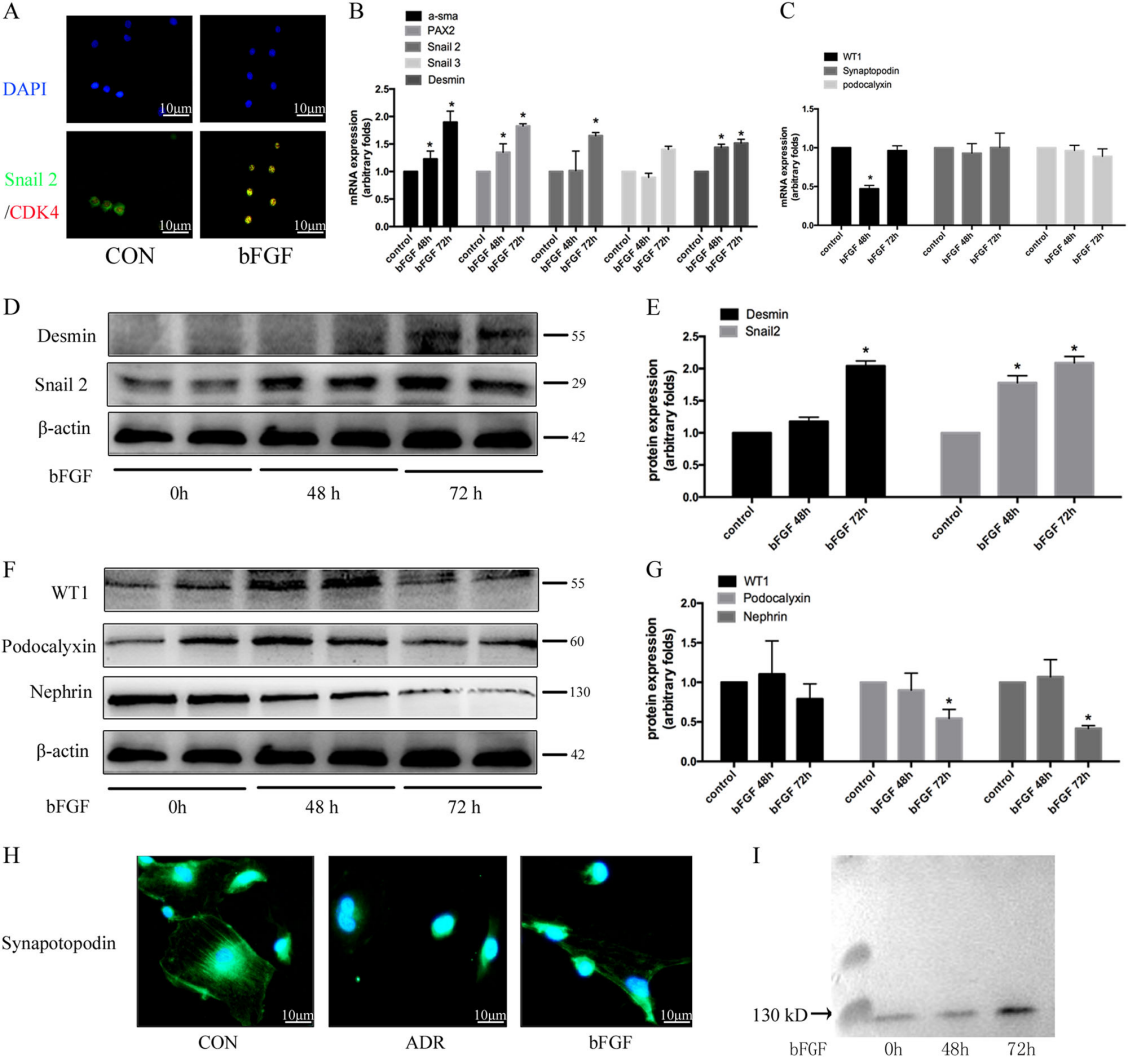
bFGF promoted podocyte dedifferentiation. **a** Immunofluorescence detection of snail2 and CDK4 expression in bFGF-treated podocytes in vitro (magnification, ×200). **b**, **c** mRNA expression of dedifferentiation-related genes and podocyte markers examined by real-time PCR after bFGF treatment for the indicated time periods. **d** The effect of bFGF on the expression of snail2 and desmin were determined by western blotting analyses. **e** Immunoblotting quantification of (D). **f** Effect of bFGF on the expression of podocyte marker proteins, examined by western blotting. **g** Corresponding graph of (F). Error bars are ± SD. (*n* = 3 per group). *P < 0.05 vs. control group. **h** Immunofluorescence staining for expression of the cytoskeleton-associated protein, synaptopodin, in adriamycin- (0.25 µg/mL) and bFGF (50 ng/mL)-treated podocytes (magnification, ×400). **i** Immunoblotting detection of nephrin shedding into the culture medium.

To further verify the relationship between re-entry into the cell cycle and podocyte dedifferentiation, we used basic fibroblast growth factor (bFGF) to induce podocytes to re-enter the cell cycle (Fig. 3a, b). After being stimulated with 50 ng/mL bFGF for 8 h and 12 h, the expression of cyclin D1 protein was up-regulated by 297.67 ± 35.57% and 318.67 ± 90.63%, respectively, while CDK4 protein expression was up-regulated by 253.53 ± 7.39% and 339.67 ± 68.39%, respectively, compared with the control group (Fig. 3c, d). As shown in Fig. 4a, in the bFGF-treated group, snail2 translocated from the cytoplasm to the nucleus. Since snail2 is an important transcription factor in the process of trans-differentiation, its transfer to the nucleus can promote the occurrence of epithelial-mesenchymal transition. In addition, compared with the control group, 72 h of bFGF treatment also up-regulated the expression of the dedifferentiation-related genes *α-SMA*, desmin, *PAX2*, and *snail2* by 97.13 ± 24.81%, 63.15 ± 4.68%, 43.73 ± 5.07%, and 72.08 ± 4.08%, respectively. At the same time, the mRNA expression of *WT1*, but not podocalyxin, synaptopodin, or nephrin, was down-regulated by 50.31 ± 5.08% initially and then returned to baseline levels after bFGF administration (Fig. 4b, c). As shown in Fig. 4d–g, the expression levels of desmin and snail2 proteins increased by 99.12 ± 4.71% and 103.52 ± 5.82%, respectively, after bFGF stimulation of cultured podocytes for 72 h, while the podocyte marker proteins, podocalyxin and nephrin, were down-regulated by 42.69 ± 10.03% and 58.14 ± 4.92%, respectively, compared with the control group. Synaptopodin is an important protein involved in the cytoskeleton of podocytes. As shown in Fig. 4h, the expression of synaptopodin decreased after adriamycin or bFGF treatment. After 48 h and 72 h of stimulation with bFGF, nephrin was detected in the cell supernatant, which may imply that nephrin was shed into the culture medium from the surface of podocytes (Fig. 4i).

### YAP signalling was activated in injured podocytes

**Fig. 5:**
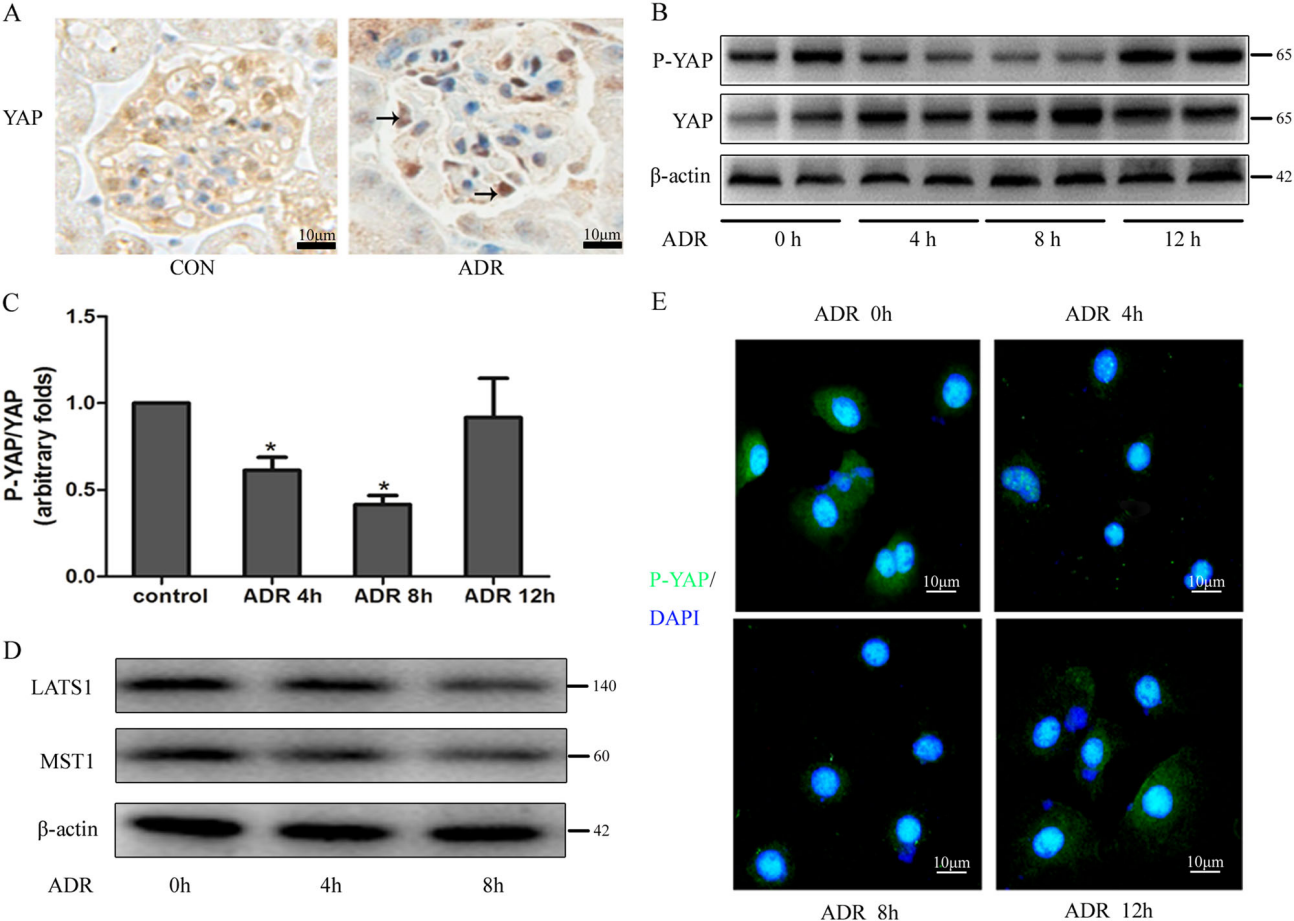
YAP signalling was activated in adriamycin-treated podocytes. **a** Immunohistochemical detection of YAP expression in mouse kidneys; podocytes are indicated by an arrow (magnification, ×400). **b** The effect of adriamycin (0.25 µg/mL) on P-YAP and YAP levels was determined by western blotting analysis. **c** Corresponding graph of (B). Error bars are ± SD. (*n* = 3 per group). *P < 0.05 vs. control group. **d** The effect of adriamycin (0.25 µg/mL) on MST1 and LATS1 levels was determined by western blotting analysis. **e** Immunofluorescence detection of P-YAP (green) levels in adriamycin-treated podocytes (magnification, 400×).

Immunohistochemical staining of mouse kidneys revealed that YAP expression was mainly located in the cytoplasm of glomerular cells in normal mice. However, in adriamycin-treated mice, high levels of YAP were observed in the nuclei of podocytes (Fig. 5a). As shown in Fig. 5b, c, phosphorylated YAP levels were down-regulated by 42.00 ± 5.0% and 60.77 ± 2.12% after 4 h and 8 h of stimulation with adriamycin, respectively, but were restored to basal levels after 12 h. This implied that adriamycin could activate the intracellular YAP signalling pathway in podocytes. We also found that adriamycin induced a decrease in the expression of the upstream YAP signalling pathway proteins, mammalian sterile 20-like kinase 1 (MST1) and Large Tumour Suppressor Kinase 1 (LATS1), thereby reducing the phosphorylation of YAP in the cytoplasm, resulting in its translocation to the nucleus (Fig. 5d). Cellular immunofluorescence analysis showed that the level of phosphorylated YAP in the cytoplasm was significantly reduced at 4 and 8 h of adriamycin administration (Fig. 5e).

### Activation of YAP signalling promoted podocyte cell cycle re-entry and subsequently, dedifferentiation

**Fig. 6:**
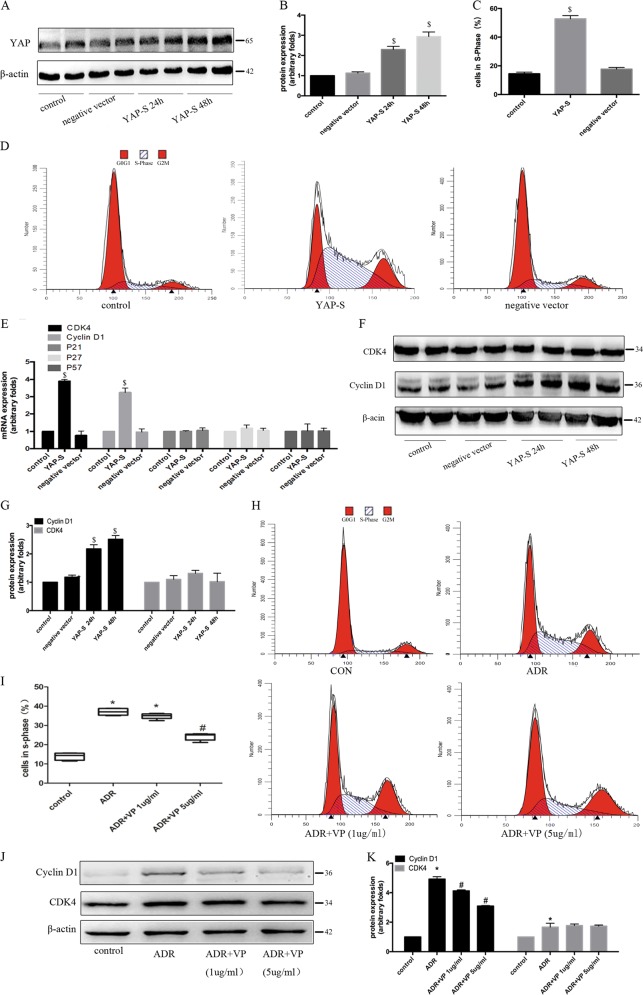
Overexpression of YAP promoted re-entry of podocytes into the cell cycle. **a** Western blotting showed up-regulation of YAP protein expression after transfection of YAP-overexpression plasmid. **b** Immunoblotting quantification of (A). YAP-S: Ser127 mutant YAP-overexpression plasmid. **c** Data show the proportion of cells in S phase in different groups of (D). **d** The effect of YAP overexpression plasmid transfection on podocyte cell cycle stage, detected by flow cytometry. **e** Real-time PCR detection of cell cycle-related gene expression after YAP overexpression. **f** The effect of YAP overexpression on CDK4 and cyclin D1 levels was determined by western blotting analysis. **g** Corresponding graph of (F). **h** Flow cytometric examination of podocyte cell cycle stage after adriamycin administration, with or without VP (1 µg/mL or 5 µg/mL) pretreatment. VP: Verteporfin. **i** Data show the proportion of cells in S phase in different groups of (H). **j**: Western blotting analysis of the effect of verteporfin on the increase in CDK4 and cyclin D1 protein levels induced by adriamycin. **k** Corresponding graph of (J). Error bars are ± SD. (*n* = 3 per group). *P < 0.05 vs. control group, ^#^*P* < 0.05 vs. adriamycin-treated group, ^$^*P* < 0.05 vs. negative control group.

To explore the effect of YAP signalling on the cell cycle of podocytes, we transfected podocytes with a plasmid expressing Ser127-mutant YAP, which cannot be phosphorylated and thus, can directly enter the nucleus to exert its transcriptional co-activation effect. At 24 and 48 h after transfection, the expression of YAP was up-regulated by 130.00 ± 15.39% and 194.01 ± 12.89%, respectively (Fig. 6a, b). After 48 h, the proportion of S-phase cells in the YAP-overexpression group was increased by 35.06 ± 1.28%, indicating that overexpression of YAP promoted the differentiation of mature podocytes and their re-entry into the cell cycle (Fig. 6c, d). As shown in Fig. 6e, g, compared with their expression in the negative control group, the expression levels of cyclin D1 and CDK4 genes in the YAP-overexpression group were 296.33 ± 5.89% and 231.07 ± 25.23% higher, respectively, while the gene expression levels of p21, p27, and p57 were not significantly different between the two groups. At the protein level, the expression of cyclin D1 was up-regulated by 100.03 ± 8.57% and 146.67 ± 7.68% after transfection of YAP plasmid for 24 and 48 h, respectively, while CDK4 protein expression showed no obvious change after YAP overexpression (Fig. 6f, g). These results suggested that YAP may promote the transition of podocytes from G1 to S phase, mainly by up-regulating the expression of cyclin D1.

Verteporfin is an inhibitor of YAP and TEAD binding and it affects the transcription factor activity of YAP^17^. As shown in Fig. 6h, i, treatment with 5 µg/ml verteporfin decreased the number of S-phase cells by 10.03 ± 1.32% compared with the adriamycin-treated group. Since verteporfin inhibited the adriamycin-induced re-entry of podocytes into the cell cycle, its effect on cell cycle-associated protein expression was further examined. As shown in Fig. 6j, k, pretreatment with verteporfin inhibited the increase in cyclin D1 expression, but not CDK4 expression induced by adriamycin.

**Fig. 7:**
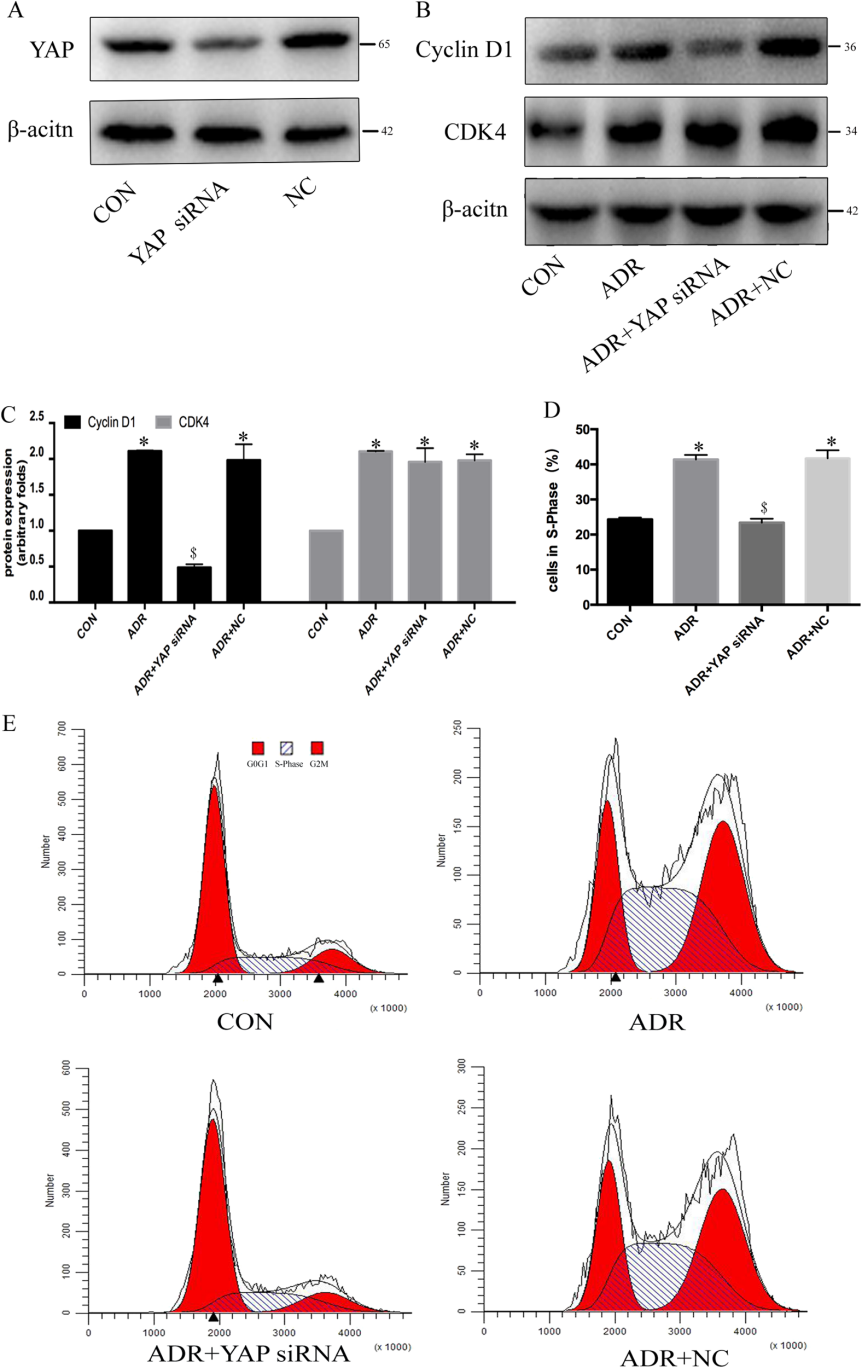
Knockdown of YAP expression inhibited podocyte re-entry into the cell cycle. **a** Western blotting showed down-regulation of YAP protein expression after siRNA interference. NC: negative control siRNA. **b** The effect of YAP siRNA interference on CDK4 and cyclin D1 levels was determined by western blotting analysis. **c** Immunoblotting quantification of **b**. **d** Data show the proportion of cells in S phase in different groups of **e**. **e** The effect of YAP siRNA on podocyte cell cycle stage, detected by flow cytometry. Error bars are ± SD. (*n* = 3 per group). *P < 0.05 vs. control group, ^$^*P* < 0.05 vs. negative control group.

To validate the above results, we then measured cyclin D1 and CDK4 protein expression after adriamycin treatment in YAP-knockdown and control podocytes. As shown in Fig. 7b, the expression of cyclin D1 was decreased by 175 ± 13.13% after knocking down YAP. However, knockdown of the YAP protein had no significant effect on the change in CDK4 protein expression induced by adriamycin. Cell cycle detection revealed that knocking down YAP reduced the proportion of cells in S phase by 20.05 ± 2.79% (Fig. 7d, e).

**Fig. 8:**
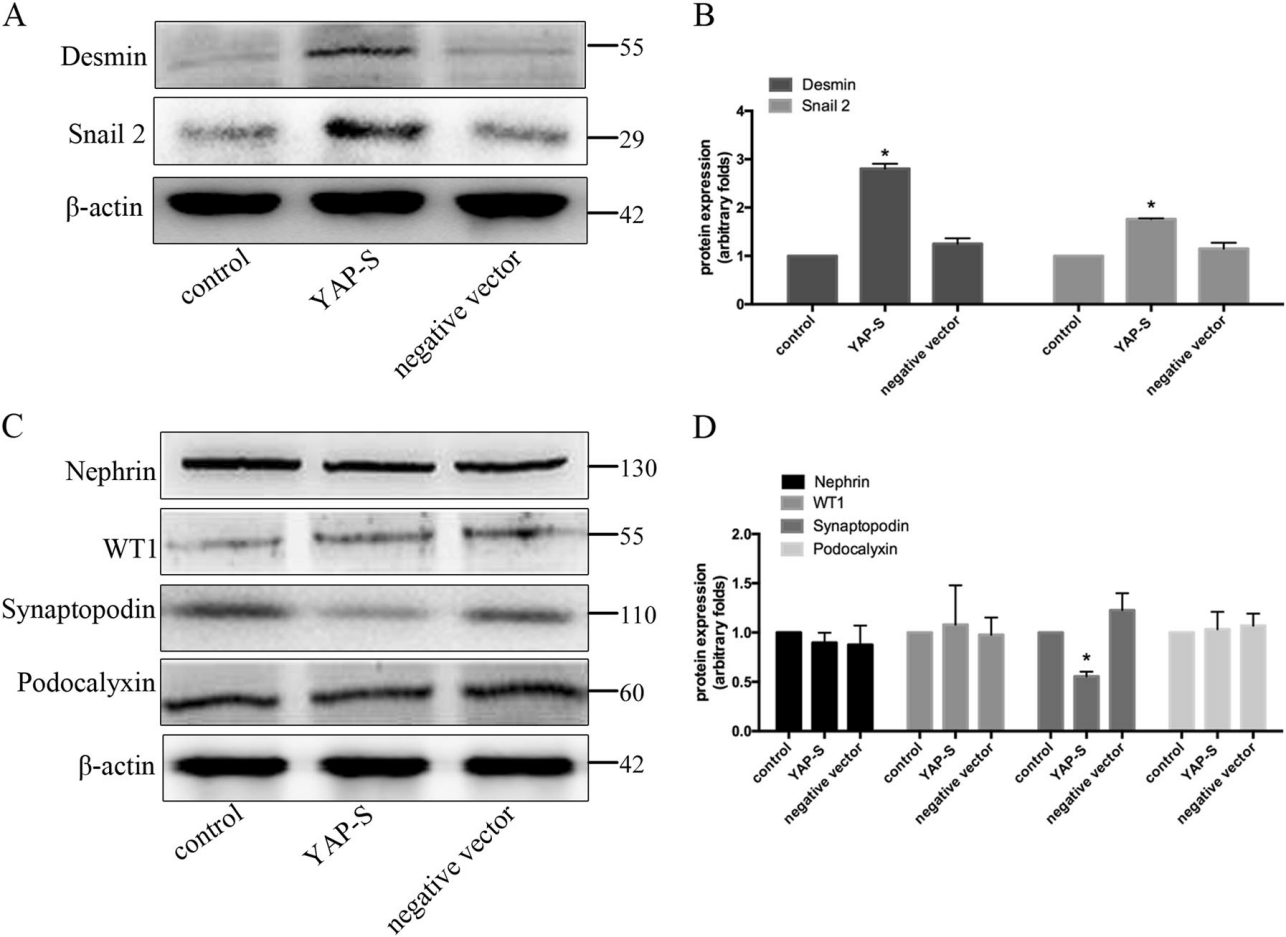
Overexpression of YAP promoted podocyte dedifferentiation. **a** The effect of YAP overexpression on the expression of snail2 and desmin proteins by western blotting. **b** Western blotting signals of **a** were quantified and total protein was normalised to the loading control, β-actin. **c**: Immunoblotting assay of the effect of YAP overexpression on podocyte marker protein expression. **d** Immunoblotting quantification of **c**. Error bars are ± SD. (*n* = 3 per group). *P < 0.05 vs. negative control group.

We found that YAP overexpression promoted the re-entry of podocytes into the cell cycle and that the induction of podocytes re-entering the cell cycle promoted their dedifferentiation. Therefore, it was necessary to further explore the effect of YAP on the dedifferentiation of podocytes. As shown in Fig. 8a, b, after transfecting podocytes with a YAP-overexpression plasmid, the expression of the dedifferentiation-related proteins, desmin and snail2, were up-regulated by 189.73 ± 5.97% and 68.56 ± 1.28% and the expression of the podocyte marker protein, synaptopodin, was down-regulated by 45.12 ± 3.78% compared to the negative control group. However, nephrin, podocalyxin, and WT1 expression levels were not significantly different among the groups (Fig. 8c, d).

## Discussion

The present study showed that adriamycin promoted the re-entry of mature podocytes into the cell cycle by up-regulating the expression of cyclin D1 and CDK4, whereas cell cycle-associated protein inhibitors were not involved in this process. Further, we found that adriamycin induced podocyte dedifferentiation. Promoting podocyte re-entry into the cell cycle with bFGF may also lead to cell dedifferentiation. Thus, cell cycle re-entry may be an important cause of dedifferentiation in podocytes. YAP played an important role in this process, since it promoted podocyte cell cycle re-entry by up-regulating the expression of cyclin D1, desmin, and snail2 proteins, thus promoting the dedifferentiation of podocytes.

Podocytes are terminally differentiated glomerular epithelial cells. During kidney development, podocyte precursor cells migrate to the glomerular capillary clusters, begin to differentiate, and are accompanied by changes in cell cycle regulation. During this period, the levels of cyclins that promote cell proliferation are reduced and the expression of cell cycle inhibitors begins to increase. These cell cycle events ultimately lead to cell cycle arrest, which is essential for maintaining the normal function and structure of mature podocytes^18^. High levels of p21, p57, and p27 expression prevent mature podocytes from overcoming the G1/S transition phase, thereby maintaining their phenotype and physiological functions^6,19^. In our study, we found that adriamycin treatment significantly increased the proportion of podocytes entering S phase of the cell cycle. Moreover, in mice with adriamycin-induced nephropathy, some podocytes began to express PCNA, which implied that some podocytes entered the cell cycle and proliferated. Recent evidence also suggests that blocking the mammalian target of rapamycin complex 1 (mTORC1)/4E-binding protein 1 (4E-BP1) signalling pathway inhibits the increases in PCNA and Ki67 expression and the fraction of primary podocytes in S phase during 2–6 h of adriamycin treatment^20^.

In human immunodeficiency virus (HIV)-associated nephropathy^21^, collapsed glomerular disease^6,22^ and cell-type Focal segmental glomerulosclerosis (FSGS)^4^, podocytes obtain the ability to proliferate. Expression of the cyclin-dependent kinase inhibitors, p27 and p57, decreased in damaged podocytes, while expression of cyclin E, cyclin A, CDK2, and cyclin B1 increased, indicating that an imbalance in cyclin-dependent kinases and their regulators drove podocytes back into the cell cycle. In the present study, the expression of cyclin D1 and the cyclin-dependent kinase, CDK4, were up-regulated, but p21 and p27 were unchanged in adriamycin-treated podocytes. Both cyclin D1 and CDK4 are major regulators that drive cells into S phase. Cyclin D1 has been shown to be important in the regulation of proliferation and differentiation^23^. It has been reported that cyclin D1 is abundant in cultured proliferating podocytes, but not in quiescent differentiated podocytes^24^. In vivo, injured podocytes show increased cyclin D1 expression in rats with passive Heymann’s nephritis and in HIV-transgenic mice, resulting in their participation in the formation of the glomerular crescent^24^. Cyclin D1 expression coincides with cell-cycle entry, although it is not necessary for the terminal differentiation of podocytes.

CDK4 is the first kinase activated by mitogenic signals. The kinase activity of CDK4 is specifically involved in progression through G1^25^. In the present study, we found that adriamycin induced an increase in the expression of CDK4 in podocytes, thereby promoting podocyte re-entry into the cell cycle. A previous study also reported that after transfecting podocytes in vitro with a CDK4-expressing lentivirus, the cells can re-enter the cell cycle and the cell phenotype significantly changes compared with normal cells. The expression of the podocyte marker molecules, synaptopodin and podocalyxin, was significantly down-regulated in this study and podocin expression was not detected^26^.

Our previous study showed that adriamycin induces podocyte apoptosis, which ultimately leads to a significant reduction in the number of podocytes^27^. Therefore, although adriamycin can induce podocytes to enter the cell cycle, it does not cause podocyte proliferation. Similarly, in other glomerular diseases, such as membranous nephropathy, a polynuclear podocyte phenotype can be found and DNA synthesis increases, as does the expression of cyclin B1 and B2, but there is no cell proliferation^28^. Because podocytes lack aurora kinase B, the cytoskeleton cannot form a spindle and therefore cannot be split into two intact cells^29^. It is unclear whether these abnormal podocytes migrate to the bare basement membrane and whether incomplete mitosis is part of the glomerular repair mechanism. Recent studies have found that the polynuclearisation of podocytes does not reduce the viability of the cells themselves, but increases their susceptibility to puromycin, suggesting that podocytes entering the cell cycle may promote cellular injury^30^. Glomerular damage leads to increased expression of cyclins in podocytes, which then induces some of these cells to enter an abnormal G2/M phase, ultimately leading to abnormal mitosis, multinucleated podocytes, and cell death, a phenomenon known as mitotic catastrophe^31^.

We further found that the expression of desmin and snail2, which are related to the dedifferentiation of podocytes, was significantly increased in the kidneys of mice with adriamycin-induced nephropathy, while podocyte maker protein expression levels were reduced, suggesting that podocytes underwent dedifferentiation, which may eventually lead to dysfunction and detachment from the basement membrane. In order to explore the relationship between re-entry into the cell cycle and cell dedifferentiation, we used bFGF to induce podocyte re-entry and found that bFGF ultimately led to podocyte dedifferentiation. The staining of kidney samples from adriamycin-treated mice showed that some CDK4-positive podocytes also began to express desmin, which indicates that the podocytes entering the cell cycle began to undergo dedifferentiation. We then speculated that adriamycin may promote podocyte re-entry into the cell cycle, combined with cell dedifferentiation.

The YAP signalling pathway was originally identified in Drosophila, where it has been shown to modulate organ size^32^. MST1/2 are key components of the Hippo signalling pathway. They combine with salvador family WW domain-containing protein 1 to form an activated complex that then initiates LATS1/2 phosphorylation^33^. Once activated, LATS1/2 further promote the signalling cascade by phosphorylating YAP at Ser127. Phosphorylated YAP is degraded in the cytoplasm, while dephosphorylated YAP can enter the nucleus and bind to TEAD1–4 to initiate transcription of downstream target genes, thereby exerting proliferation and anti-apoptosis effects^34^. YAP signalling is involved in the pathogenesis of cystic nephropathy and is up-regulated in fibroblasts to increase the synthesis of the extracellular matrix and to promote renal fibrosis^35^. A comparison of our data with two independent studies of transcriptional regulation in human FSGS glomeruli obtained from the Nephroseq database found that focal segmental glomerulopathy is also associated with an activation of the Hippo pathway in podocytes^36^. Further studies have found that the loss of YAP in podocytes leads to podocyte apoptosis^37,38^. In the present study, we found that adriamycin down-regulated the expression of MST1 and LATS1, thereby allowing dephosphorylated YAP to accumulate in podocytes. The overexpression of activated YAP in podocytes promoted podocyte re-entry into the cell cycle, because cyclin D1, but not CDK4, was significantly up-regulated in the podocytes overexpressing activated YAP. In fact, cyclin D1 has been confirmed as a target gene downstream of YAP and the inhibition of YAP expression can significantly reduce the protein levels of intracellular cyclin D1^39^. The present study also found that pretreatment with verteporfin prevented the YAP-induced increase in cyclin D1 expression. Moreover, knockdown of YAP significantly inhibited the adriamycin-induced upregulation of cyclin D protein expression. Together, these data suggested that YAP may promote the transition of podocytes from G1 to S phase, mainly by up-regulating the expression of cyclin D1.

At present, most studies on YAP in podocytes suggest that the activation of YAP signalling has a protective effect on podocytes. Podocyte-specific YAP knockout can lead to the loss of foot processes, impaired renal function, and proteinuria^36,40^. In the present study, we found that the expression of desmin and snail2 significantly increased in podocytes overexpressing YAP, while expression of the podocyte cytoskeleton-associated protein, synaptopodin, significantly decreased after YAP overexpression/ Therefore, we propose that the activation of YAP signalling may have promoted podocyte re-entry into the cell cycle, leading to changes in the cytoskeleton and podocyte dedifferentiation. In a rat model of puromycin nephropathy, high levels of YAP expression and significantly increased mRNA levels of the YAP/TAZ downstream target genes *Cyr61*, *Annkrd1*, *Ctgf*, and *Diaph3* are observed in the glomerulus 2 days after the model is established. Furthermore, it was shown that the expression of extracellular matrix components, such as collagen COL6A1 and its receptor, BCAM, or the profibrotic matrix metalloproteinase, ADAMTS1, significantly increased after podocyte overexpression of YAP and that YAP signalling activation and fibrosis are closely related. Proteinuria occurs in transgenic mice overexpressing YAP, whereas administration of verteporfin inhibits the progression of proteinuria in puromycin-treated rats^41^. Therefore, early blocking of YAP signalling activation may be an important potential strategy for preventing podocyte injury.

In conclusion, we found that YAP signalling up-regulated the expression of podocyte dedifferentiation-associated proteins. Thus, we propose that YAP signalling is involved in the regulation of adriamycin-induced podocyte cell cycle regulation and dedifferentiation. Although there are reports that YAP can be used as an anti-apoptotic target to protect podocytes, our results suggested that the activation of YAP signalling in the early stages of cell damage was detrimental to maintaining the phenotype and normal biological function of podocytes.

## Materials and methods

### Experimental drugs

Adriamycin was purchased from Sigma Chemical (St. Louis, MO, USA), Recombinant murine bFGF was purchased from PEPROTECH (Rocky Hill, NJ, USA), Verteporfin was purchased from Tocris Bioscience (Bristol, UK).

### Podocyte culture

Conditionally immortalised mouse podocytes were kindly provided by Peter Mundel and were cultured, as described previously^42^. The majority of the analysed cells had an arborous shape and expressed synaptopodin. All experiments were repeated at least three times for each indicated condition. Podocytes between passages 9 and 20 were used in all experiments.

### Urine albumin/creatinine ratio

Urine albumin and creatinine concentrations were determined using an albumin and creatinine assay kit (Jiancheng, Nanjing, China). Absorbance was determined at 510 nm using a microplate reader.

### Immunofluorescence and immunohistochemical staining

Cryosections with a thickness of 4 μm were prepared using a cryostat and were fixed in 4% paraformaldehyde for 15 min. After blocking, the cryosections were incubated with primary antibodies and then with a fluorescein Cy3-FITC-labelled secondary antibody (1:100; Proteintech, Wuhan, China). Fluorescence images were recorded using a TCS SP5II confocal microscope (Leica, Bensheim, Germany). The following primary antibodies were used: anti-desmin (1:100; Santa Cruz Biotechnology, Inc., Dallas, TX, USA), anti-podocalyxin (1:100; R&D Systems, Minneapolis, MN, USA), and anti-snail2 (1:100, Proteintech). Podocytes were seeded onto clean glass coverslips, fixed with 4% paraformaldehyde, and permeabilised with 0.2% Triton X-100. The slides were incubated with an anti-PCNA (1:100, Proteintech), anti-synaptopodin (1:100; Proteintech), anti-CDK4 (1:100; Abcam, Cambridge, UK), anti-P-YAP (1:100; Cell Signaling Technology, Danfoss, MA, USA), or anti-snail2 (1:100, Proteintech) antibody.

For immunohistochemistry analysis, after deparaffinisation, rehydration, antigen retrieval, and blocking, the sections were incubated with an anti-PCNA (1:100), anti-CDK4 (1:100), anti-desmin (1:100), anti-YAP (1:100, Proteintech), or anti-cyclin D1 (1:100, Cell Signaling Technology) primary antibody and then with a horseradish peroxidase-labelled secondary antibody (Beyotime, Shanghai, China).

### RNA extraction and real-time PCR

Total RNA was extracted using TRIzol (Thermo Scientific, Waltham, MA, USA), according to the manufacturer’s instructions. After reverse transcription, cDNA samples were denatured and amplified using a LightCycler 480 real-time PCR system (Roche Applied Science, Mannheim, Germany). Amplification conditions were as follows: 45 cycles of 95 °C for 30 s, 95 °C for 10 s, and 60 °C for 20 s. The primers used are shown in Table 1.

**Table 1:** Oligonucleotides used in real-time PCR for the selected genes.

Gene product	Upstream sequence (5’-3’)	Downstream sequence (5’-3’)	Length (bp)
GAPDH	CCAATGTGTCCGTCGTGGATCT	GTTGAAGTCGCAGGAGACAACC	233
P21	CCTGGTGATGTCCGACCTG	CCATGAGCGCATCGCAATC	103
P27	TCAAACGTGAGAGTGTCTAACG	CCGGGCCGAAGAGATTTCTG	103
P57	CGAGGAGCAGGACGAGAATC	GAAGAAGTCGTTCGCATTGGC	118
CyclinA	GCAGGCTGTGGCTTACTAGG	GATTGCTGTGATCTCCTGGC	202
CyclinB1	AGCGAAGAGCTACAGGCAAG	CTCAGGCTCAGCAAGTTCCA	141
CyclinB2	CCGACGGTGTCCAGTGATTT	CTGAGGTTTCTTCGCCACCT	144
CyclinD1	GCGTACCCTGACACCAATCTC	CTCCTCTTCGCACTTCTGCTC	183
CyclinD2	GAGTGGGAACTGGTAGTGTTG	CGCACAGAGCGATGAAGGT	154
CyclinE1	GTGGCTCCGACCTTTCAGTC	CACAGTCTTGTCAATCTTGGCA	101
CyclinE2	ATGTCAAGACGCAGCCGTTTA	GCTGATTCCTCCAGACAGTACA	198
CDK1	AGAAGGTACTTACGGTGTGGT	GAGAGATTTCCCGAATTGCAGT	128
CDK2	CCTGCTTATCAATGCAGAGGG	TGCGGGTCACCATTTCAGC	203
CDK4	ATGGCTGCCACTCGATATGAA	TCCTCCATTAGGAACTCTCACAC	129
ZO-1	GCTTTAGCGAACAGAAGGAGC	TTCATTTTTCCGAGACTTCACCA	156
WT1	TCTTCCGAGGCATTCAGGATG	TGCACACATGAAAGGACGTTT	101
Synaptopodin	AGCAAGTGAAAGAAGCAAAGTCT	CAGTACCGTAACTGACTAGGGT	144
Nephrin	CAGCGATGATGCGGAGTACG	CAGCTACCCAGGTAACTGTGC	149
Desmin	GTGGATGCAGCCACTCTAGC	TTAGCCGCGATGGTCTCATAC	218
Snail2	TGGTCAAGAAACATTTCAACGCC	GGTGAGGATCTCTGGTTTTGGTA	131
Snail3	CACACGCTGCCTTGTGTCT	GGTCAGCAAAAGCACGGTT	133
α-sma	GTCCCAGACATCAGGGAGTAA	TCGGATACTTCAGCGTCAGGA	102
PAX2	AAGCCCGGAGTGATTGGTG	CAGGCGAACATAGTCGGGTT	101

### RNA interference

YAP small interfering RNA (siRNA) and a negative-control siRNA were purchased from Jiman Company (Shanghai, China). siRNAs (100 nmol/L) were transiently transfected using Lipofectamine RNAiMax (Thermo Scientific), according to the manufacturer’s instructions.

### Immunoblotting analysis

Protein was extracted from fractionated cells the concentration was determined using a bicinchoninic acid reagent (Thermo Scientific). For cell supernatant protein extraction, the cell supernatant was collected, centrifuged at 12,000 × *g* for 10 min at 4 °C, and 20 μL of the cell supernatant was aspirated from near the bottom of the centrifuge tube, taking care not to touch the cell pellet. Protein samples were resolved by SDS–PAGE and transferred to a nitrocellulose membrane. Immunoblot bands were visualised using a Tanon imaging system (Zhejiang, China). The antibodies used were: anti-p21/p27 (1:500; Santa Cruz Biotechnology, Inc.), anti-synaptopodin (1:500, Proteintech), anti-cyclin D1 (1:1000, Cell Signaling Technology), anti-CDK4 (1:1000, Abcam), anti-YAP (1:1000, Cell Signaling Technology), anti-P-YAP (1:1,000, Cell Signaling Technology), anti-snail2 (1:500, Proteintech), anti-desmin (1:500, Proteintech), anti-ZO-1 (1:500, Proteintech), anti-MST1 (1:1,000; Cell Signaling Technology), anti-LATS1 (1:1,000, Cell Signaling Technology), anti-WT1 (1:500, Proteintech), anti-nephrin (1:1000, Abcam), and anti-β-actin (1:500, Proteintech).

### Animal experiments

All animal experiments were performed using a protocol approved by Renji Hospital, Shanghai Jiaotong University School of Medicine. Male specific-pathogen-free BALB/c mice (Shanghai SLAC Laboratory Animal, Shanghai, China) aged 6 to 8 weeks were divided into four groups: a control group and an adriamycin treatment group. Nephropathy was induced by a single intravenous injection of 10 mg/kg (of body weight) of adriamycin. Urine was collected for 24 h using a metabolic cage on days 0, 4, 8, and 16. At day 16, mice were sacrificed under chloral hydrate anaesthesia and the kidneys were removed for subsequent analyses. The animal experiment was approved by Ren Ji Hospital Committee and complied with ethical regulations.

### Flow cytometric cell cycle analysis

Cell cycle assays were performed using a Cell Cycle and Apoptosis Analysis Kit (Beyotime Biotechnology, Shanghai, China), according to the manufacturer’s instructions. Finally, red fluorescence was detected by flow cytometry (FACS Vantage SE; Becton Dickinson, Franklin Lakes, NJ, USA) at an excitation wavelength of 488 nm. Light scattering was detected concurrently and plotted using FlowJo software (Becton Dickinson).

### Plasmid construction and transfection

A plasmid directing the overexpression of Ser127 mutant YAP, which cannot be phosphorylated, was purchased from Jiman. A fragment of the target gene was first amplified using the following primers: forward, 5′-CAAGCTGGCTAGCGTTTAAACGG-3′ and reverse, 5′-GTAGTCGGATCCTAACCACGTGAGAAAG-3′. The amplified fragment was then ligated into am adenoviral vector (PDC316-mCMV-ZsGreen1-CMV-MCS-3Flag) through restriction sites contained at both ends. The ligation product was then transfected into competent bacterial cells. Newly grown monoclonal colonies were sequenced to confirm their identity and by aligning the correct clones, the successfully constructed overexpressing adenoviral vector containing the gene of interest was constructed. Plasmid transfection was performed using a FuGENE transfection kit (Promega, Madison, WI, USA), according to the manufacturer’s instructions.

### Statistical analyses

Quantitative data are representative of at least three experiments. The results are expressed as mean ± standard deviation (SD). Statistical analyses were performed using SPSS v19.0 software (IBM, Chicago, IL, USA). An analysis of variance, followed by Duncan’s test and Dunnett’s test, were used to assess differences between multiple groups. *P* values < 0.05 were considered statistically significant.
